# Stress resilience in *Coffea arabica* and *Coffea canephora* under harsh drought and/or heat conditions: selected genes, proteins, and lipid integrated responses

**DOI:** 10.3389/fpls.2025.1623156

**Published:** 2025-07-24

**Authors:** José C. Ramalho, Isabel Marques, Isabel P. Pais, Jean Armengaud, Duarte Gouveia, Ana P. Rodrigues, Danielly Dubberstein, António E. Leitão, Miroslava Rakočević, Paula Scotti-Campos, Sónia Martins, Magda C. Semedo, Fábio L. Partelli, Fernando C. Lidon, Fábio M. DaMatta, Ana I. Ribeiro-Barros

**Affiliations:** ^1^ Plant-Environment Interactions and Biodiversity Lab (PlantStress & Biodiversity), Forest Research Center (CEF), Associate Laboratory TERRA, School of Agriculture, University of Lisbon (ISA/ULisboa), Lisboa, Portugal; ^2^ Unidade de Geobiociências, Geoengenharias e Geotecnologias (GeoBioTec), Faculdade de Ciências e Tecnologia (FCT) Universidade NOVA de Lisboa (UNL), Caparica, Portugal; ^3^ Unidade de Investigação em Biotecnologia e Recursos Genéticos, Instituto Nacional de Investigação Agrária e Veterinária, I.P. (INIAV), Oeiras, Portugal; ^4^ Université Paris-Saclay, CEA, INRAE, Département Médicaments et Technologies pour la Santé (DMTS), Bagnols-sur-Cèze, France; ^5^ Centro Universitário do Norte do Espírito Santo (CEUNES), Dept. Ciências Agrárias e Biológicas (DCAB), Universidade Federal Espírito Santo (UFES), São Mateus, ES, Brazil; ^6^ Assistência Técnica e Gerencial em Cafeicultura - Serviço Nacional de Aprendizagem Rural (SENAR), Porto Velho, RO, Brazil; ^7^ Setor de Fisiologia Vegetal, Laboratório de Melhoramento Genético Vegetal, Centro de Ciências e Tecnologias Agropecuárias, Universidade Estadual do Norte Fluminense (UENF), Campos dos Goytacazes, Rio de Janeiro, Brazil; ^8^ Área Departamental de Engenharia Química, Instituto Superior de Engenharia de Lisboa, Instituto Politécnico de Lisboa, Lisboa, Portugal; ^9^ Deptartamento de Biologia Vegetal, Universidade Federal Viçosa (UFV), Viçosa, MG, Brazil

**Keywords:** antioxidant response, climate change, coffee, drought, heat, membrane lipid dynamics, proteomic and transcriptional profiles, stress superimposition

## Abstract

Climate change has intensified the frequency, severity, and simultaneous incidence of drought and heat events, threatening the sustainability of agricultural systems worldwide. This implies the use of resilient plant genotypes able to activate defense mechanisms and overcome stress damage. We examined the leaf transcriptomic, proteomic, and membrane lipid responses in two cultivars of the main coffee-producing species—*Coffea arabica* L. cv. Icatu and *Coffea canephora* Pierre ex A. Froehner cv. Conilon Clone 153 (CL153—subjected to single and combined exposure to severe water deficit (SWD) and heat (up to 42°C/30°C, day/night). Well-watered (WW) plants maintained under adequate temperature (25°C/20°C) were gradually exposed to SWD and afterward to a slow temperature increase up to 42°C/30°C, followed by a 2-week recovery (Rec14) after reestablishing temperature and water conditions. Gene regulation and the respective protein contents were often marginally correlated; however, CL153 and, especially, Icatu showed markedly greater abundance of transcripts and/or proteins of most molecules to the imposed stress conditions, along with altered lipid profiles of chloroplast membranes. A set of key complementary response mechanisms, expressed either commonly or in a genotype- or stress-dependent manner, was identified. Additionally, the common responses to all stress conditions reflected stress crosstalk and interaction. Drought (with or without heat superimposition) constituted a greater response driver than heat in both genotypes. These showed *de-novo* synthesis of lipids and proteins, altering the fatty acid profile and unsaturation degree of chloroplast membranes and strengthening oxidative stress protection. The latter involved several genes and their respective proteins (e.g., aquaporins, PIPs and TIPs; chaperonins, Chape 20 and 60; dehydrin, DH1; dehydration-responsive element binding protein, DREB1D-F1; early light-induced protein, ELIP; heat shock protein 70 kDa, HSP70; ascorbate peroxidases, APXs; catalase, CAT), particularly prominent in Icatu. Also, a major recovery was found, although several genes/proteins exhibited lasting effects by Rec14. Overall, we revealed newly shared and specific (genotype or stress) responses of a complex network supporting *Coffea* spp. resilience. The identification of reliable stress-responsive traits is crucial to ensure the sustainability of this important tropical crop facing future climate stress scenarios, in which superimposed drought and heat stresses will be more frequent.

## 1 Introduction

Since the industrial revolution in the 18th century, atmospheric greenhouse gas concentrations, such as CO_2_, NH_4_, and N_2_O, have greatly increased due to anthropogenic activities, leading to perceptible increases in air temperature and changes in temporal and regional rainfall distribution patterns (van Beek et al., 2010; Cassia et al., 2018). Although recent estimates suggest that vegetation has been gradually acclimating to the new warming conditions and that the negative impacts of climate change on terrestrial ecosystem productivity may be less severe than previously assumed (Fang et al., 2024), constraints related to temperature (cold/heat), water (drought/waterlogging), and salinity are gradually impacting agriculture, with declines in yield to less than half in major crops, due to sensitivity of growth, development, and reproductive processes (Wang et al., 2003; Fábián et al., 2019; Balfagón et al., 2020; Pais et al., 2023; Oliveira et al., 2024). Exceeding thermal stress thresholds can significantly reduce vegetation productivity and C-uptake at a global scale (Li et al., 2024), with expected stronger impacts in tropical latitudes where plants evolved with narrower thermal ranges (Harrington et al., 2016; Li et al., 2024).

In C3 plants, rising temperatures affect all major physiological processes, stimulating photorespiration and mitochondrial respiration to a greater extent than photosynthesis (Ainsworth and Rogers, 2007). Additionally, chloroplasts are among the first affected structures (Mano, 2002), since heat inactivates photosystem (PS) II (electron acceptor and donor sides), impairs electron transport, reduces RuBisCO activity (Crafts-Brandner and Salvucci, 2000; Haldimann and Feller, 2004; Balfagón et al., 2020), alters protein structure, promotes the formation of highly reactive molecules of oxygen (ROS) and chlorophyll, and interferes with essential transcriptional and translational processes (Wahid et al., 2007; Song et al., 2014; Dusenge et al., 2019). Heat also modifies hormone and primary and secondary metabolite balance (Jamloki et al., 2021), and by stimulating an overfluidization of cell membrane lipids, it potentially disrupts membrane-based processes, specifically in chloroplasts (Wahid et al., 2007).

Under drought, stomata closure is among the first responses even under mild severity, reducing water loss by transpiration, but also limiting latent heat loss (increasing leaf temperature) and CO_2_ diffusion to chloroplast carboxylation sites (Menezes-Silva et al., 2017; DaMatta et al., 2024). Under severe drought, non-stomatal limitations to photosynthesis will take place at the photochemical and biochemical levels (Chaves et al., 2003; Fahad et al., 2017). The lower photochemical energy use can secondarily prompt oxidative conditions, boosting ROS formation that additionally damages lipids and proteins in the photosynthetic apparatus (Halliwell, 2006; Wahid et al., 2007; Osakabe et al., 2014).

Single stress factors activate signaling pathways that regulate specific gene expression, protein synthesis, and metabolite production, supporting plant defense responses, which limit damage and enhance resilience (Potters et al., 2007; Jaspers and Kangasjarvi, 2010; Fernandes et al., 2021; Marques et al., 2022a).

However, the co-occurrence of environmental stressors is increasingly frequent, namely heat and water deficit. Though a prior mild drought exposure could increase plant physiological tolerance to a subsequent heat stress (Ramalho et al., 2018; Araújo et al., 2019; Zhang et al., 2019), the superimposition of these stresses usually aggravates single stress impacts on mineral balance and on the morphological, physiological, metabolic, and gene expression levels (Pandey et al., 2015; Dubberstein et al., 2020), particularly on C-assimilation, greatly depressing plant growth and crop yields and ultimately compromising plant survival (Lamaoui et al., 2018; Balfagón et al., 2020). Notably, it was recently shown that warming per se, by increasing the atmospheric evaporative demand, amplifies drought severity by an average of 40% globally, hitting typically dry regions but also wet areas (Gebrechorkos et al., 2025).

Each stress combination triggers unique responses in gene expression, metabolism, and nutrient assimilation and balance, different from the addition of responses promoted by single stresses (Way et al., 2015; Zandalinas et al., 2018; Balfagón et al., 2020). Such specific and shared responsive signaling pathways and processes, constituting a complex and interconnected network that crosstalks at several levels (Fujita et al., 2006; Yamaguchi-Shinozaki and Shinozaki, 2006; Balfagón et al., 2020), act in a coordinated and dynamic manner, often with genotype- and stress-specific responses (Han et al., 2024), ultimately governing acclimation (Pandey et al., 2015; Zandalinas et al., 2021). At the molecular level, plants respond to high temperatures and drought by triggering complex signaling networks that include stress perception, transduction cascades (e.g., via calcium signaling, ROS, and phytohormones like ABA), and activation of stress-responsive genes (Sato et al., 2024). These include transcription factors (e.g., *DREB*, *NAC*, *HSF* families), molecular chaperones (e.g., *HSP70*), dehydrins, and antioxidative enzymes (e.g., ascorbate peroxidases, catalase, superoxide dismutases) that mitigate oxidative damage and promote cellular homeostasis (Trono and Pecchioni, 2022; Aina et al., 2024; Marques et al., 2024). Aquaporins (*PIPs* and *TIPs*) play key roles in regulating water transport, with dehydration or heat often modulating their expression and abundance in a stress-dependent manner (Ahmed et al., 2021). In addition, the reorganization of membrane lipids and the enhancement of protective pigments (e.g., zeaxanthin, via VDE activity) support membrane integrity preservation and photosynthetic efficiency (Qiao et al., 2024). Therefore, understanding plant response mechanisms that specifically prevent, mitigate, and/or counteract stress impacts and support plant performance under concurrent stress conditions is therefore vital to select and breed crops that maintain quality and yield production (Rodrigues et al., 2016; Balfagón et al., 2020; Zandalinas et al., 2021; Qiao et al., 2024).

Among the 131 species of the *Coffea* genus so far described (Royal Botanic Gardens, 2025), *Coffea arabica* L. (Arabica coffee) and *Coffea canephora* Pierre ex A. Froehner (Robusta coffee) support the coffee value chain, currently accounting for ca. 57% and 43% of the world yield, respectively. Despite some price volatility and production uncertainties, global coffee production for 2024/2025 is projected to reach approx. 10.572 million tons (Embrapa, 2024), with an estimated income of ca. USD 256,000 million (Precedence Research, 2024). The coffee value chain involves over 12.5 million farms, with ca. 60% owned by smallholders (Koutouleas et al., 2022); contributes to the livelihoods of ca. 25 million smallholder farmers in approximately 80 producing countries in the tropical region (DaMatta et al., 2019; Pham et al., 2019); and involves between 60 and 125 million people worldwide (Sachs et al., 2019).

The two main coffee-producing species are perennial woody plants of the Rubiaceae family, which have quite similar cultivation requirements but distinct temperature and precipitation needs. *Coffea arabica* thrives better in a milder climate, requiring a mean annual temperature of approximately 18°C to 23°C (with tolerance up to 24°C–25°C) and well-distributed rainfall throughout the year (preferably above 1,600 mm). In turn, *C. canephora* needs an annual rainfall of ~1,800 mm but tolerates higher mean temperatures of 22°C to 26°C, or even warmer, being considered more heat- and less cold-tolerant than *C. arabica* (DaMatta et al., 2018, 2024).

Heat and drought are major environmental constraints that hamper coffee plant growth, productivity, and quality (DaMatta and Ramalho, 2006). Despite coffee cultivation having made considerable headway, with significant technological and scientific advances in production and quality, predictions of future global climate conditions indicate severe constraints to its sustainability, including yield declines, loss of adequate areas, and altered pest and disease incidence (Magrach and Ghazoul, 2015; van der Vossen et al., 2015; Pham et al., 2019). As coffee plantations can last for more than 30 years, such impacts may be further exacerbated as the actual plantations will endure increasingly harsher climate conditions (Bunn et al., 2015). Although leaf thermal tolerance seems to be closely dependent on leaf age (Vilas-Boas et al., 2024), some elite cultivars (particularly those selected for full sun cropping) show greater resilience to environmental stresses than traditionally assumed (DaMatta et al., 2018; Dubberstein et al., 2020; Semedo et al., 2021; Rodrigues et al., 2024).

*Coffea* plants are able to trigger a wide and coordinated set of defense responses to single environmental stresses. These include altered gene expression, increased levels of photoprotective and antioxidative components (e.g., pigments and enzymes) and other molecules (e.g., thylakoid electron carriers involved in cyclic electron flow), and adjustments in the lipid matrix of chloroplast membranes (Ramalho et al., 2000; Pinheiro et al., 2004; Martins et al., 2016; Scotti-Campos et al., 2019; Marques et al., 2021; Semedo et al., 2021; DaMatta et al., 2018). Genes associated with drought tolerance in *C. canephora* and *C. arabica* include TFs (e.g., DREB-like genes) and ROS control, i.e., coding for *SOD*s and *APX*s (Marraccini et al., 2012; Vieira et al., 2013; Torres et al., 2019; Fernandes et al., 2021). In addition, heat greatly upregulates the expression of genes related to protective molecules (Martins et al., 2016; Marques et al., 2021). Yet, drought and heat combination promotes more complex responses than the sum of individual stresses in *Coffea* spp (Marques et al., 2024). Given the abovementioned facts, this study aims to deepen our understanding of the complex network of complementary defense mechanisms triggered in cropped genotypes of the two main *Coffea* species, to identify key resilience features to single and combined severe drought and heat stresses. Thus, we will look simultaneously into the changes in the patterns of gene expression and the abundance of key selected proteins (e.g., aquaporins, HSP70), as well as chloroplast membrane lipid profile dynamics, all involved in plant stress acclimation.

## 2 Materials and methods

### 2.1 Plant material and environmental conditions

Two cropped genotypes of *C. canephora* Pierre ex A. Froehner cv. Conilon Clone 153 (CL153) and *C. arabica* L. cv. Icatu Vermelho (an introgressed variety resulting from a cross of *C. canephora* and *C. arabica* cv. Bourbon Vermelho, then further crossed with *C. arabica* cv. Mundo Novo) were evaluated following an experimental design previously set (Dubberstein et al., 2020). Briefly, 32 plants in total were grown for 7 years in 80 L pots in two walk-in growth chambers (EHHF 10000, ARALAB, Portugal), in a substrate consisting of a mixture of soil, peat, and sand (3:1:3, v/v/v), with pH 6.5, and under controlled temperature (25°C/20°C, day/night, ± 1°C), PAR irradiance (ca. 700–800 μmol m^−2^ s^−1^, at the upper canopy level), relative humidity (70% ± 2%), photoperiod (12 h), and air [CO_2_] (380 ± 5 μL CO_2_ L^−1^).

Irradiance was provided by a combination of fluorescent (Lumilux L58W/840, Osram, Germany) and halogen (100 W, Halolux Ceram, Osram) lamps. Plants were fertilized (see Ramalho et al., 2013) and well-watered (WW) by adequate irrigation every 2 to 3 days. Water deficit and heat conditions were sequentially imposed in a gradual manner in order to allow plant acclimation (see below), in eight plants per treatment and genotype.

### 2.2 Imposition of severe drought conditions

Water conditions were first imposed under adequate temperature (25°C/20°C), considering the exposure to approx. 80% (WW) or 10% (severe water deficit, SWD) of maximal pot water availability, exactly as described earlier (Dubberstein et al., 2020; Rodrigues et al., 2024). For that, WW plants were kept fully irrigated (predawn water potential, *Ψ*_pd_ ≥ −0.35 MPa; relative water content, RWC ≥ 91%), while SWD plants were subjected to a partial withholding irrigation (through a partial reposition of water that was lost in each pot, every 2 days) for 2 weeks to promote SWD conditions (reaching values of *Ψ*_pd_ below −3.7 MPa and of RWC close to 60%). After this, the SWD plants were maintained under these conditions for another 5 days before evaluation at the control 25°C/20°C temperature, as well as during the entire exposure to increased temperature (see 2.3).

### 2.3 Imposition of high temperature and reestablishment of initial conditions

The mentioned WW and SWD conditions were maintained along a gradual temperature increase of 0.5°C day^−1^ (of diurnal temperature) from 25°C/20°C up to 42°C/30°C, with stabilization of 5 days at 31°C/25°C, 37°C/28°C, and 42°C/30°C to enable the programmed evaluations, exactly as described in Rodrigues et al. (2024). Finally, temperature was readjusted to 25°C/20°C, and then all the plants were irrigated to the initial watering conditions (80% of maximal pot water availability), and their potential recovery was monitored for 14 days (Rec14).

### 2.4 Water status monitoring

Leaf *Ψ*_pd_ was measured using a pressure chamber (Model 1000, PMS Instrument Co., Albany, OR, USA), according to Schölander et al. (1965), in individual leaves of five or six plants per treatment and genotype. Samplings every 2 or 3 days allowed a close monitoring, though only data from key points during temperature increase and recovery periods were shown.

Leaf RWC was estimated as described for *Coffea* spp (Ramalho et al., 2018), using 10 foliar discs of 0.5 cm^2^ each, punched from the same leaves used for *Ψ*_pd_ determinations. RWC values (%) were calculated as = [(FW − DW)/(TW – DW)] × 100, where FW represents the fresh weight determined immediately after cutting the discs, TW is the turgid weight obtained after overnight rehydration of the discs in a humid chamber at ca. 20°C, and DW is the dry weight obtained after drying the discs at 80°C for 48 h.

Both RWC_pd_ and *Ψ*_pd_ measurements were performed at predawn on five to six replicates per treatment, every 2 to 3 days, but only the data at the main temperature points for data samplings (considering temperature rise and both heat and drought recoveries) were presented.

### 2.5 Sampling and processing

Samplings were made using newly matured leaves from the upper (well-illuminated) part of six to eight plants per treatment and genotype, under photosynthetic steady-state conditions (after ca. 2 h of light) at 25°C/20°C (control), 37°C/28°C, 42°C/30°C, and Rec14. The same leaf pool per plant was used for all evaluations. The freshly collected leaf material was immediately used for lipid analysis or flash frozen in liquid N_2_ and stored at –80°C, which was finely powdered in liquid N_2_ prior to protein and gene expression analysis. Leaf tissue extractions were performed using an ice-cold mortar and pestle and cold homogenizing solutions.

### 2.6 Lipid profiling of chloroplast membranes

#### 2.6.1 Lipid extraction from chloroplast membranes

Enriched chloroplast membrane fractions were obtained from ca. 4 g FW of leaf tissue, as optimized for *Coffea* spp (Scotti-Campos et al., 2014, 2019). Briefly, freshly cut leaf material was homogenized in 25 mL of a cold 50 mM MES buffer (pH 6.4), containing 0.4 M of D-sorbitol, 10 mM of NaCl, 5 mM of MgCl_2_, 2 mM of EDTA, 1 mM of MnCl_2_, 0.4% (w/v) BSA, and 2 mM of Na-ascorbate. The homogenate was filtered through eight layers of cheesecloth and centrifuged (3,000*g*, 5 min, 4°C). For lipid extraction, the obtained chloroplast pellet was mixed with 9 mL of a chloroform/methanol/water (1/1/1, v/v/v) solution and centrifuged (4,500*g*, 10 min, 4°C). The lower chloroform phase was selected and evaporated to dryness under N_2_ flux, and the lipid residue was resuspended in 1.5 mL of an ethanol:toluene (1:4) mixture, for further use in the next steps.

#### 2.6.2 Total fatty acid analysis

For fatty acid (FA) analysis, a 50-μL aliquot of the lipid resuspension was saponified and methylated with BF_3_. To quantify FAs, heptadecanoic acid (C17:0) was added to each sample as an internal standard. FA methyl esters (FAME) were analyzed using GC-FID (Varian, CP-3380, Agilent Technologies, Santa Clara, CA, USA), with a DB-Wax capillary column 0.25 mm i.d. × 30 m, 0.25 μm (J&W Scientific, Agilent Technologies, Santa Clara, CA, USA). Column temperature was programmed to rise from 80°C to 200°C at 12°C min^−1^, after 2 min at the initial temperature. The injector and detector temperatures were 200°C and 250°C, respectively. The carrier gas was hydrogen with a flow rate of 1 mL min^−1^, at a split ratio of 1:50 of the sample. Individual FAs were identified by comparison with a standard mixture (FAME Mix, Restek, Bellefonte, PA, USA). Total FAs (TFAs) denote the sum of individual FAs. TFA unsaturation degree was calculated as the double bond index (DBI = [(%monoenes + 2 × %dienes + 3 × %trienes)/%saturated FAs]), following Mazliak (1983).

### 2.7 Gene expression studies

Total RNA was extracted following Marques et al. (2024) using the Analytik-Jena InnuSPEED Plant RNA Kit (Analytik Jena Innuscreen GmbH, Jena, Germany). cDNA was synthesized from 1 μg of total RNA using the SensiFAST™ cDNA Synthesis kit (Meridian Bioscience, Cincinnati, OH, USA), according to the manufacturer’s recommendations. PCR reactions were prepared using the SensiFAST™ SYBR No-ROX kit (Meridian Bioscience) following the protocol and the parameters described in Marques et al. (2023). Reactions were carried out in 96-well plates using a qTOWER 2.2 Thermal Cycler (Analytik, Jena, Germany) using the following parameters: hot start activation of the Taq DNA polymerase at 95°C for 10 min, followed by 40 cycles of denaturation at 95°C for 15 s, annealing at 60°C for 30 s, and elongation at 72°C for 30 s. A melting curve analysis was performed at the end of the PCR run by a continuous fluorescence measurement from 55°C to 95°C with sequential steps of 0.5°C for 15 s. A single peak was obtained, and no signal was detected in the negative controls. Three technical replicates were performed. Expression studies included 14 selected genes (**Supplementary Tables S1**; **S2**), namely, aquaporins (*CaPIP2*, *CaTIP2*), dehydrin (*DH1a*), dehydration-responsive element-binding protein 1D (*DREB1D-F1*), chloroplast 70 kDa heat shock-related protein (*HSP70*), chloroplast early light-induced protein (*ELIP*), chloroplast 20 kDa chaperonin (*Chape 20*), mitochondria chaperonin CPN60 (*Chape 60*), antioxidative enzymes [*e.g*., catalase (*CAT*), cytosolic (*APXCyt*), and chloroplast ascorbate (*APXChl*) peroxidases], and violaxanthin de-epoxidase (*VDE2*). Malate dehydrogenase (*MDH*) and ubiquitin (*UBQ10*) were used as reference genes (Martins et al., 2017). This selection of genes (and most corresponding proteins, as shown below) was based on prior evidence of involvement in abiotic stress responses (drought and/or heat), with emphasis on those known to play roles in oxidative stress protection, membrane stabilization, water transport, and stress signaling pathways. Specifically, aquaporins (PIP and TIP families), chaperonins, dehydrins, heat shock proteins, antioxidative enzymes (APXs, CAT, SOD), and regulatory transcription factors (DREB1D-F1) were targeted. Candidate genes were identified from *Coffea* spp. transcriptomic databases and previous studies (e.g., Fernandes et al., 2021; Marques et al., 2022a, b) and validated by expression profiles under similar stress conditions.

### 2.8 Protein abundance evaluation

Protein extraction from ca. 200 mg FW of powdered frozen leaves, liquid chromatography and high-resolution mass spectrometry (NanoLC-MS/MS) peptide analysis, and protein identification and quantification were carried out as described in Dubberstein et al. (2020). The reference database from *C. canephora* (Denoeud et al., 2014) downloaded on 1 July 2019 was used for peptide and protein inference by the MASCOT Daemon 2.6.1 search algorithm (Matrix Science). A targeted approach was used, selecting a set of 17 proteins (**Supplementary Table S3**) usually involved in plant stress responses and closely associated with the selected genes (see 2.5.2). Protein annotation was obtained from the UniProt Knowledgebase (UniProtKB) (https://www.uniprot.org/uniprot/?query=&sort=score). Mass spectrometry proteomics data were deposited in the ProteomeXchange Consortium via the PRIDE partner repository, with the dataset identifier PXD019474 and DOI: 10.6019/PXD019474 for the *C. arabica* proteome and the dataset identifier PXD019541 and DOI: 10.6019/PXD019541 for the *C. canephora* proteome.

### 2.9 Experimental design and statistical analysis

Plants of CL153 and Icatu genotypes were independently subjected to eight treatment combinations, forming a 2 × 4 factorial consisting of two water availability levels (WW or SWD) and four levels of temperature (25°C/20°C, 37°C/28°C, 42°C/30°C, and Rec14) under a completely randomized design, with eight plants per treatment and genotype. Altogether, the entire experiment lasted 82 days: SWD plants reached the desired *Ψ*_pd_ within 14 days upon gradual drought imposition and were kept in these conditions another 5 days before the temperature was increased to 42°C/30°C (49 days). Soil was later fully watered and the temperature was set to 25°C/20°C, and plants were analyzed for stress relief for 14 days (Rec14).

Datasets were analyzed using a two-way ANOVA to assess differences between water availability levels, temperature treatments, and their interaction. Mean comparisons (independently for each genotype) were conducted using Tukey’s HSD test. Statistical analyses were performed in STATISTICA v7.0 (StatSoft, Hamburg, Germany) with a 95% confidence level applied to all the tests.

## 3 Results

### 3.1 Imposed leaf water conditions

Water restriction resulted in SWD, assessed by *Ψ*_pd_ mean values of ca. −3.9 (CL153) and −3.7 MPa (Icatu) at 25°C/20°C, with concomitant values of RWC close to 60% in both genotypes (**Table 1**). In contrast, though single heat (at the two highest temperatures) doubled *Ψ*_pd_ values, no significant changes occurred in water status in WW plants at 42°C/30°C, when they reached −0.8 MPa (CL153) and −0.7 MPa (Icatu). The good hydration status of WW plants at the highest temperature was further confirmed by the RWC value, which was maintained at 95% in both genotypes.

**Table 1:** Variation of leaf water potential (*Ψ*_pd_) and relative water content (RWC_pd_) determined at pre-dawn in *Coffea canephora* cv. Conilon Clone 153 (CL153) and *Coffea arabica* cv. Icatu plants submitted to well-watered (WW) and severe water deficit (SWD), followed by a temperature increase from 25°C/20°C (day/night) to 42°C/30°C and a 14-day recovery period after stress relief (Rec14).

Genotype	Water	Temperature (day/night)																			
		25°C/20°C				31°C/25°C				37°C/28°C				42°C/30°C				Rec14			
		*Ψ*_pd_ (MPa)																			
**CL153**	**WW**	−0.30	±	0.05	aA	−0.54	±	0.03	aA	−0.64	±	0.17	aA	−0.77	±	0.24	aA	−0.66	±	0.07	aA
	**SWD**	−3.85	±	0.44	bB	−3.56	±	0.23	bB	−4.47	±	0.31	bB	−4.38	±	0.15	bB	−0.93	±	0.11	aA
**Icatu**	**WW**	−0.35	±	0.02	aA	−0.51	±	0.07	aA	−0.55	±	0.03	aA	−0.70	±	0.12	aA	−0.49	±	0.05	aA
	**SWD**	−3.69	±	0.19	bB	−4.60	±	0.32	bC	−4.46	±	0.33	bC	−4.14	±	0.45	bC	−0.54	±	0.09	aA
		RWC (%)																			
**CL153**	**WW**	91.1	±	1.2	aA	93.7	±	0.8	aA	92.0	±	1.5	aA	94.6	±	0.6	aA	90.8	±	1.3	aA
	**SWD**	57.5	±	3.7	bB	62.1	±	2.6	bB	59.1	±	2.2	bB	57.5	±	3.2	bB	88.9	±	1.5	aA
**Icatu**	**WW**	96.2	±	1.1	aA	92.5	±	0.6	aA	94.5	±	1.1	aA	95.3	±	0.5	aA	92.4	±	0.8	aA
	**SWD**	60.7	±	2.5	bB	52.6	±	1.7	bC	51.7	±	3.2	bC	47.3	±	1.1	bC	89.9	±	1.8	aA

For each parameter, different letters after the mean values ± SE (*n* = 5–6) express significant differences between temperature treatments for the same water level (A, B, C, D) or between water availability levels for each temperature treatment (a, b), always separately for each genotype.

The combined stress exposure led to further *Ψ*_pd_ decline, to minimal values close to −4.5 MPa at 37°C/28°C in both cultivars (not significantly different from the values at 42°C/30°C), together with a greater dehydration only in Icatu-SWD plants that reached minimal RWC values below 50% at 42°C/30°C.

Notably, after the simultaneous restoration of water and temperature control conditions, SWD plants of both genotypes showed almost complete recoveries of *Ψ*_pd_ and RWC from 4 days onward up to 2 weeks (Rec14), especially in WW plants, as compared to their respective controls.

### 3.2 Altered expression of selected genes related to stress protection

**Figure 1:**
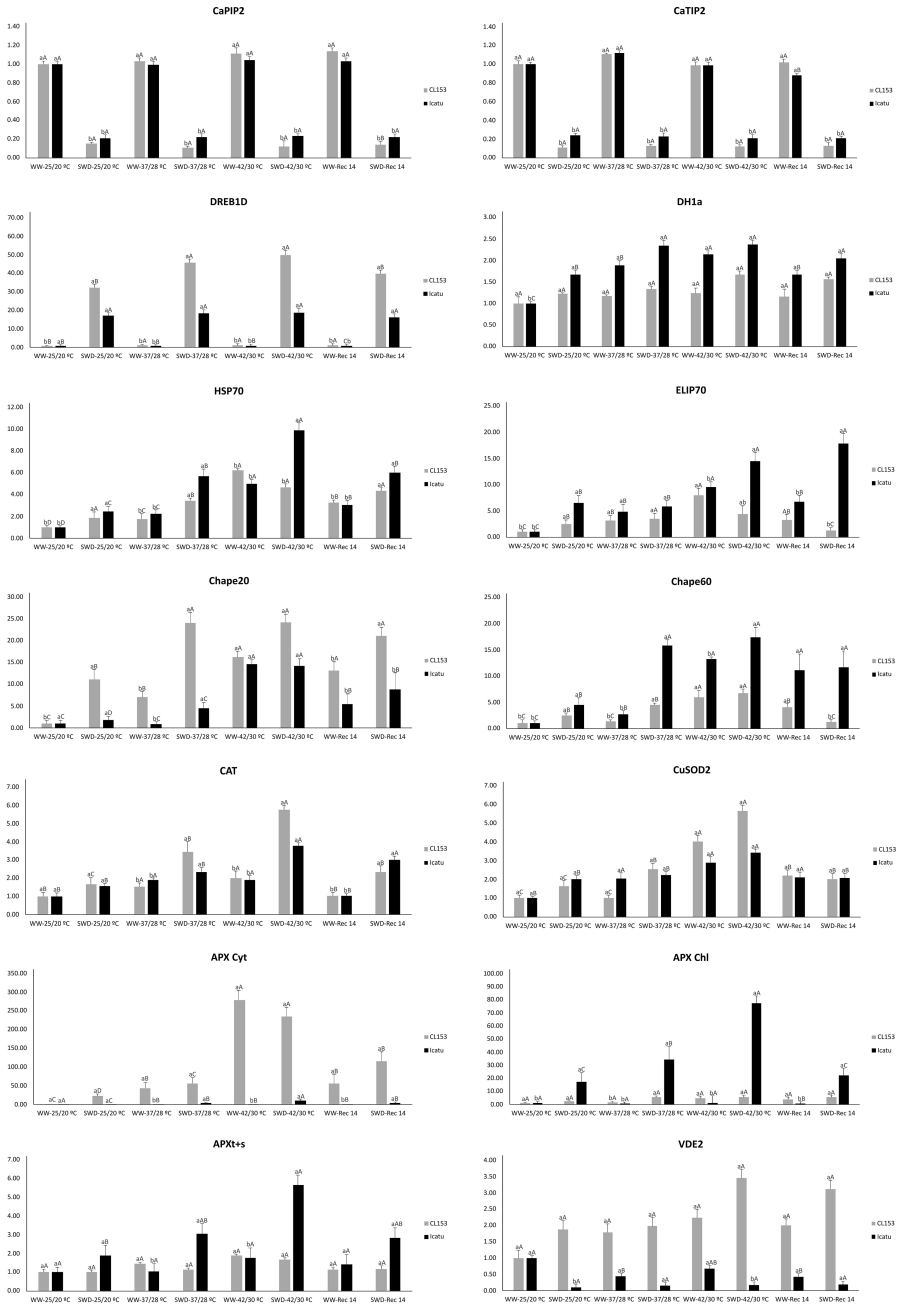
Expression of genes associated with stress response mechanisms in *Coffea canephora* cv. Conilon Clone 153 (CL153) and *Coffea arabica* cv. Icatu plants submitted to well-watered (WW) and severe water deficit (SWD), followed by a temperature increase from 25°C/20°C (day/night) to 42°C/30°C and a 14-day recovery period after stress relief (Rec14). The RT-qPCR gene expression values represent the *n*-fold relative to the double control (25°C/20°C, WW) within each genotype. Original expression values for each gene resulted from the mean ± SE (*n* = 6), from three independent biological assays. Different letters express significant differences between temperature treatments for the same water level (A, B, C, D) or between water availability levels for each temperature treatment (a, b), always separately for each genotype (numerical values and statistical analysis can be found in **Supplementary Table S2**).

A strong impact of the imposed water and/or temperature conditions was found in selected genes (**Figure 1**; **Supplementary Table S2**) coding for proteins associated with plant stress response. In both genotypes, genes coding for intrinsic proteins of the plasma membrane (*CcPIP2*) and tonoplast (*CcTIP2*) subfamilies of aquaporins, as well as the dehydration-responsive element-binding transcription factor gene (*DREB1D-F1*), were unresponsive to heat. In contrast, these genes were highly sensitive to drought (regardless of temperature), distinctly downregulated (aquaporins) or upregulated (*DREB1D-F1*), a pattern sustained by Rec14. Notably, in CL153, the combined stress exposure (SWD at 37°C/28°C and 42°C/30°C) further amplified the expression of *DREB1D-F1* found in SWD plants at 25°C/20°C.

The dehydrin DH1a gene exhibited minimal responsiveness, if any, to all stress conditions in CL153, whereas in Icatu, a somewhat higher expression was observed under single heat stress (37°C/28°C and 42°C/30°C) or combined with SWD.

In general, genes coding for three chloroplast proteins (stroma heat shock, *HSP70*; early light-induced protein, *ELIP*; 20 kDa chaperonin, *Chape 20*), along with the mitochondrial chaperonin CPN60 (*Chape 60*) were slightly but systematically upregulated by single drought in both genotypes, particularly in Icatu (except for Chape 20). Heat stress alone (37°C/28°C and, especially, 42°C/30°C) promoted an even greater upregulation of these genes. That was somewhat further amplified under the combined stress exposure (SWD, 37°C/28°C) concerning *Chape 20* in CL153 and *HSP70*, *ELIP*, and *Chape 60* in Icatu. The Chape 20 gene was one of the few genes with a stronger response in CL153 than Icatu, especially under SWD and combined stresses. Although with a decline by Rec14, both WW and SWD plants often had higher expression than in their initial controls, especially for *ELIP* and *Chape 60* in Icatu, as well as *HSP70* and *Chape 20* in both genotypes.

Among the genes coding for enzymes directly involved in ROS control, the transcript abundance of catalase isozyme 1 (*CAT*), Cu,Zn-superoxide dismutase (*CuSOD_2_*), peroxisomal ascorbate peroxidase (*APXt+s*), and violaxanthin de-epoxidase (*VDE2*) barely responded to a single drought in either genotype. Still, a strong gene upregulation was found for cytosol ascorbate APX (*APXCyt*) in CL153 and chloroplast APX (*APXChl*) in Icatu. In contrast, heat alone consistently upregulated these genes, strongly in CL153, with *APXCyt* standing out with the greatest value among all the studied genes in this genotype. Notably, stress superimposition (SWD, 42°C/30°C) prompted an even greater expression of *CAT* in CL153 and of all the genes in Icatu (except *VDE2*). Such Icatu overresponse was particularly strong in *APX* genes, especially in *APXChl*, which is associated with chloroplast antioxidative protection. Finally, it was noteworthy that, as noted for several of the abovementioned genes, the upregulation was usually maintained by Rec14, but to a lower extent than at 42°C/30°C.

### 3.3 Changes in the abundance of proteins associated with stress response

**Figure 2:**
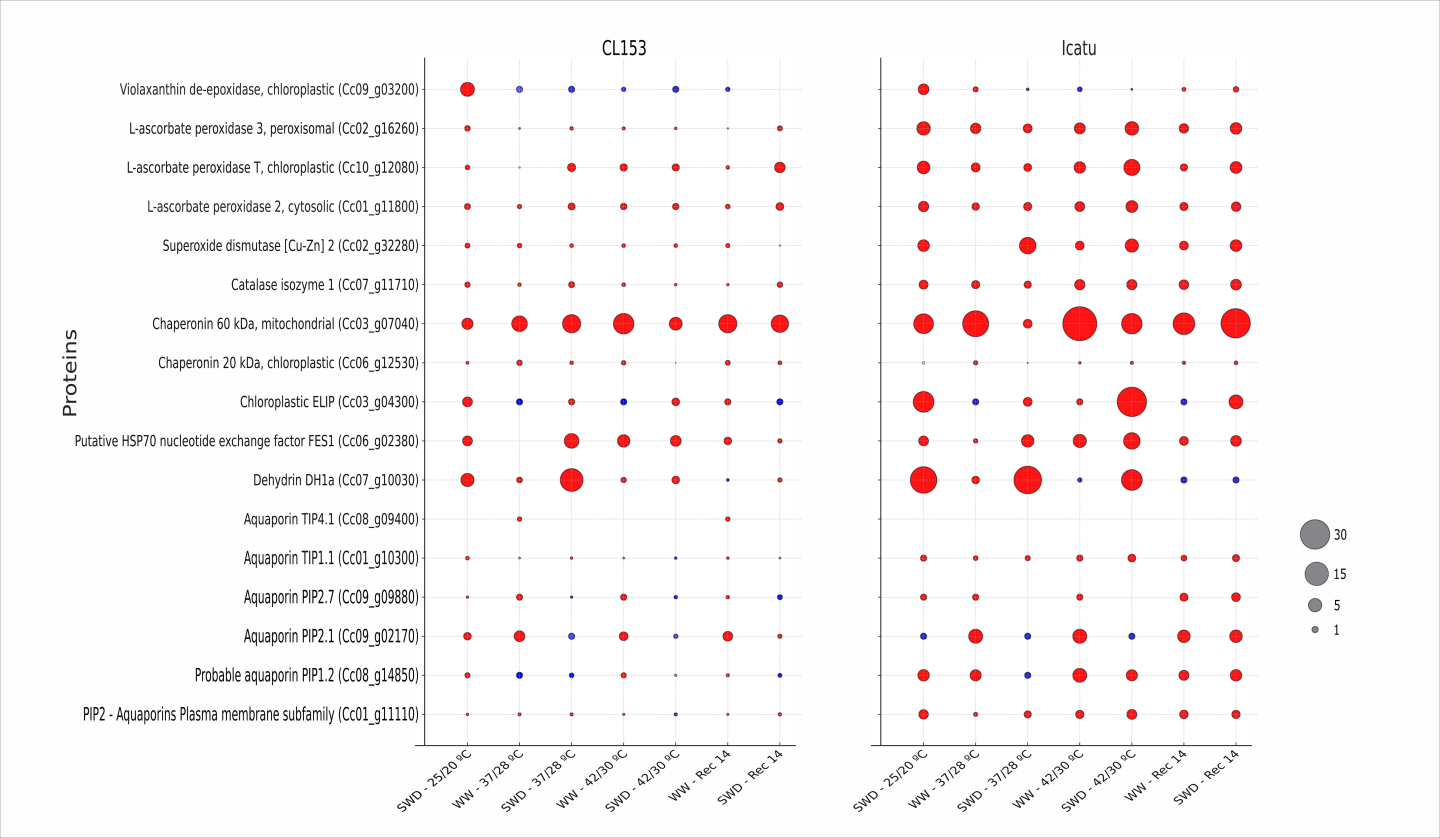
Bubble chart with the relative change of abundance of proteins related to stress response mechanisms in *Coffea canephora* cv. Conilon Clone 153 (CL153) and *Coffea arabica* cv. Icatu plants submitted to well-watered (WW) and severe water deficit (SWD), followed by a temperature increase from 25°C/20°C (day/night) to 42°C/30°C and a 14-day recovery period after stress relief (Rec14). Colors indicate an increased (red) or decreased (blue) abundance of proteins within each genotype, whereas the size of the circles reflects the extent of the variation. Values represent the mean (*n* = 3) from three independent biological assays (numerical values and statistical analysis can be found in **Supplementary Table S3**).

As referred above, most selected proteins were coded by the presented genes so that transcriptomic and proteomic patterns of changes could be integrated (**Figure 2**; **Supplementary Table S3**). The intrinsic protein subfamilies of AQPs from plasma (PIP) and tonoplast (TIP) membranes showed different changes, both between subfamilies and among individual members within each subfamily.

Overall, AQPs were more responsive to drought in Icatu, although CL153 denoted a higher constitutive level (25°C/20°C; WW plants). In detail, PiP2 protein abundance significantly increased in Icatu under both single drought and heat (42°C/30°C). In CL153, no changes were observed in either condition, but it should be noted that there was a higher constitutive value along the entire experiment (including Rec14), similar to the maximal values found in Icatu. Among the other protoplasm AQPs (PIP1.2, PIP2.1, PIP2.7), protein abundance usually tended to rise under the single stresses in both genotypes, although significant only for PIP1.2 in Icatu. The opposite was observed under stress superimposition (SWD, 42°C/30°C) as compared with the single stresses (except for PIP2 in Icatu).

The tonoplast aquaporin TIP1.1 showed a significant rise in CL153 only under single drought, but although without statistical significance, it seems noteworthy to mention that Icatu showed a double abundance under both single SWD and 42°C/30°C and even greater with stress combination. TIP4.1 was undetected in most treatments, regardless of genotype.

Dehydrin DH1a protein abundance was not impacted by single heat stress, but greatly increased under SWD conditions in both genotypes, at all temperatures and with a positive interaction at 37°C/28°C in CL153. In Rec14, this protein returned to control values.

Among the group of protective proteins in chloroplasts (HSP70, ELIP, and Chape 20) and mitochondria (Chape 60), Chape 20 showed a particularly high constitutive abundance (WW, 25°C/20°C) in both genotypes. This protein was also one of the few that showed a higher value or response pattern to single drought and heat conditions in CL153 than in Icatu plants. All the abovementioned chloroplast proteins tended to have higher values in response to single drought or 42°C/30°C (except ELIP), although it was non-significant. In contrast, the mitochondria Chape 60 greatly accumulated under either single stress in both genotypes, more prominently under heat in Icatu (with ca. 30-fold increase). Notably, ELIP markedly increased in Icatu under the stress combination, in comparison to the respective WW plants at 42°C/30°C. By Rec14, only Chape 20 and 60 maintained increased levels in WW and SWD plants.

In contrast with the small gene expression changes, the abundance of enzymes associated with oxidative stress control (CAT, APX Cyt, APX Chl, APXt+s, and VDE) was among the most responsive ones to drought or heat in both genotypes, particularly in Icatu. The exception was Cu/ZnSOD_2_, which showed moderate increases in response to drought and/or heat in both genotypes, whereas VDE abundance increased only under SWD (greatly in Icatu). Also, only Icatu showed an additional abundance increase of all APX proteins under the stress combination and maximal values for CAT and APXs in all stress conditions. Except Cu/ZnSOD_2_ and VDE, increased levels of all of these enzymes were maintained by Rec14, in both genotypes, especially in SWD plants.

### 3.4 Chloroplast membrane lipid dynamics

A quantitative FA analysis showed that the TFA content of chloroplast membranes was mostly unaffected in CL153 plants, regardless of the imposed stress conditions (**Table 2**). This contrasted with Icatu showing significant TFA increments due to drought (68% at 25°C/20°C), heat (47% at 37°C/28°C), and stress combination (142% under SWD by 37°C/28°C), although by 42°C/30°C, these values declined, approaching those found at 25°C/20°C. Also, contrasting patterns were observed among genotypes by Rec14, with higher (Icatu) and lower (CL153) TFA values than at the initial WW-25°C/20°C conditions.

**Table 2:** Evaluation of lipid dynamics of chloroplast membranes associated with total fatty acids (TFAs) and the individual fatty acids [palmitic acid (C16:0), palmitoleic acid (C16:1*c*+*t*), stearic acid (C18:0), oleic acid (C18:1), linoleic acid (C18:2), and linolenic acid (C18:3)], as well as the resulting double bond index (DBI), in *Coffea canephora* cv. Conilon Clone 153 (CL153) and *Coffea arabica* cv. Icatu plants submitted to well-watered (WW) and severe water deficit (SWD), followed by a temperature increase from 25°C/20°C (day/night) to 42°C/30°C and a 14-day recovery period after stress relief (Rec14).

Genotype	Water	Temperature (day/night)																			
		25°C/20°C				31°C/25°C				37°C/28°C				42°C/30°C				Rec14			
		TFA (mg g^−1^ DW)																			
**CL153**	**WW**	22.46	±	0.79	aA	19.34	±	0.61	aAB	19.82	±	1.34	aAB	22.31	±	1.34	aA	16.75	±	1.22	aB
	**SWD**	23.24	±	0.56	aA	20.31	±	2.54	aAB	18.51	±	0.11	aAB	19.36	±	0.10	aB	17.32	±	0.36	aB
**Icatu**	**WW**	14.13	±	0.52	bC	17.04	±	0.27	bBC	20.80	±	0.12	bAB	14.93	±	0.77	bC	22.16	±	2.49	aA
	**SWD**	23.75	±	1.12	aBC	28.34	±	0.18	aB	34.16	±	1.45	aA	20.10	±	0.20	aC	22.13	±	0.51	aC
		DBI																			
**CL153**	**WW**	6.30	±	0.40	bAB	4.85	±	0.32	bAB	6.99	±	0.57	aA	4.55	±	0.13	aB	5.19	±	0.21	bAB
	**SWD**	9.14	±	0.75	aA	6.42	±	1.05	aB	6.33	±	0.25	aB	5.81	±	0.02	aB	7.64	±	0.12	aAB
**Icatu**	**WW**	3.57	±	0.34	bB	4.90	±	0.07	bA	5.75	±	0.05	aA	3.50	±	0.26	bB	3.85	±	0.02	bB
	**SWD**	7.42	±	0.34	aA	6.11	±	0.06	aB	4.97	±	0.19	aC	5.96	±	0.02	aB	4.97	±	0.11	aC
		C16:0 (% mol)																			
**CL153**	**WW**	22.43	±	1.16	aBC	27.06	±	1.65	aAB	20.62	±	1.42	aC	28.93	±	1.63	aA	25.76	±	0.33	aABC
	**SWD**	18.08	±	1.41	aA	20.88	±	2.54	aA	20.65	±	0.53	aA	22.91	±	0.11	aA	18.97	±	0.30	bA
**Icatu**	**WW**	33.87	±	1.18	aA	26.57	±	0.29	aBC	25.50	±	0.06	aC	33.90	±	2.13	aA	29.66	±	0.01	aB
	**SWD**	21.80	±	0.88	bB	23.13	±	0.18	aB	25.50	±	0.60	bB	22.15	±	0.26	bB	27.34	±	0.38	aA
		C16:1*c*+*t* (% mol)																			
**CL153**	**WW**	3.34	±	0.55	aA	2.79	±	0.30	aA	1.04	±	0.27	bB	1.76	±	0.15	bAB	2.05	±	0.31	aAB
	**SWD**	2.80	±	0.08	aA	3.20	±	0.77	aA	3.41	±	0.24	aA	3.60	±	0.09	aA	2.96	±	0.01	aA
**Icatu**	**WW**	2.42	±	0.29	aB	3.43	±	0.30	aA	0.18	±	0.05	bC	2.00	±	0.15	bB	3.76	±	0.02	aA
	**SWD**	3.13	±	0.04	aBC	3.87	±	0.12	aAB	3.34	±	0.20	aABC	4.05	±	0.10	aA	2.68	±	0.06	bC
		C18:0 (% mol)																			
**CL153**	**WW**	7.61	±	0.25	aA	8.45	±	0.59	aA	7.86	±	0.28	aA	8.33	±	1.14	aA	8.04	±	0.55	aA
	**SWD**	5.30	±	0.15	bA	9.58	±	0.78	aA	8.59	±	0.45	aA	8.55	±	0.05	aAB	7.30	±	0.21	bB
**Icatu**	**WW**	8.26	±	1.04	aAB	8.70	±	0.15	aA	6.53	±	0.17	bB	8.50	±	0.24	aA	9.92	±	0.13	aA
	**SWD**	5.16	±	0.10	bC	6.89	±	0.09	bBC	8.72	±	0.33	aA	8.17	±	0.15	aAB	7.09	±	0.19	bAB
		C18:1 (% mol)																			
**CL153**	**WW**	1.76	±	0.02	aB	2.71	±	0.27	aA	2.10	±	0.03	bB	2.70	±	0.06	bA	2.74	±	0.06	aA
	**SWD**	2.25	±	0.07	aC	1.73	±	0.15	bD	6.21	±	0.05	aA	3.65	±	0.02	aB	2.63	±	0.02	aC
**Icatu**	**WW**	2.41	±	0.18	aC	1.61	±	0.02	bD	2.38	±	0.04	bC	3.16	±	0.04	bB	3.95	±	0.02	aA
	**SWD**	2.11	±	0.02	aC	3.65	±	0.04	aA	5.16	±	0.08	aA	5.05	±	0.06	aA	3.73	±	0.01	aB
		C18:2 (% mol)																			
**CL153**	**WW**	11.43	±	0.20	aAB	11.15	±	0.56	aAB	11.15	±	0.07	aAB	10.01	±	0.16	aB	13.75	±	0.11	aA
	**SWD**	8.41	±	1.30	aA	10.15	±	1.70	aA	8.42	±	0.05	aA	8.42	±	0.03	aA	9.36	±	0.01	bA
**Icatu**	**WW**	14.86	±	0.87	aA	11.22	±	0.14	aB	14.58	±	0.09	aA	15.27	±	0.55	aA	13.66	±	0.03	aAB
	**SWD**	9.09	±	0.08	bB	11.47	±	0.08	aAB	10.51	±	0.08	bAB	10.25	±	0.08	bAB	12.81	±	0.04	aA
		C18:3 (% mol)																			
**CL153**	**WW**	53.43	±	1.38	bA	47.85	±	1.07	aA	57.23	±	1.62	aA	48.27	±	0.56	aA	47.62	±	0.86	bA
	**SWD**	63.15	±	0.42	aA	54.46	±	5.88	aAB	52.73	±	0.94	aB	52.87	±	0.08	aB	58.78	±	0.31	aAB
**Icatu**	**WW**	38.18	±	3.14	bB	48.46	±	0.48	aA	50.83	±	0.26	aA	37.18	±	1.24	bB	39.05	±	0.09	bB
	**SWD**	58.71	±	0.85	aA	50.98	±	0.21	aB	46.77	±	0.94	aB	50.34	±	0.09	aB	46.35	±	0.47	aB

For each parameter, different letters after the mean values ± SE (*n* = 3) express significant differences between temperature treatments for the same water level (A, B, C, D) or between water availability levels for each temperature treatment (a, b), always separately for each genotype.

The qualitative changes were assessed through FAs’ relative weight and their unsaturation degree. DBI rise reflected significant increases in FA unsaturation under single drought, in both genotypes, whereas single heat increased DBI at 37°C/28°C only in Icatu. However, a DBI decline was found from 37°C/28°C to 42°C/30°C, together with higher DBI values in SWD plants at the maximal imposed temperature (as compared with WW counterparts), in both genotypes. By Rec14, the values tended to those of the initial control in both genotypes. Such DBI modifications mainly resulted from the opposite changes in the relative weight of the major saturated palmitic (C16:0) and the highly unsaturated linolenic (C18:3) acids, which together accounted for ca. 75% (CL153) and 72% (Icatu) of the chloroplast membranes’ TFAs.

The most represented FA, C18:3, significantly increased upon both single drought and heat (37°C/28°C) imposition in Icatu, while in CL153, it rose under SWD and was unresponsive to heat. Still, under 42°C/30°C, the C18:3 values in WW plants were similar to those of plants at control temperature in both genotypes. The stress combination at maximal temperature reduced C18:3 as compared to the SWD value at 25°C/20°C, but these were still higher than in WW plants at 42°C/30°C, especially in Icatu. By Rec14, the C18:3 values approached those of the initial control, but SWD plants of both genotypes kept greater C18:3 values than their WW counterparts, similar to what happened due to SWD impact alone at 25°C/20°C.

The second most represented FA, C16:0, declined under single drought and showed a stress interaction (SWD, 42°C/30°C) in both genotypes, being significant only in Icatu. Single heat significantly reduced C16:0 in Icatu plants up to 37°C/28°C (but not at 42°C/30°C), whereas in CL153, an increase was found only at the highest temperature. By Rec14, these FA values were close to the initial control conditions in WW plants of both genotypes, but the SWD ones maintained lower values than the WW counterparts (significant in CL153).

The third and fourth most represented FAs, the unsaturated linoleic acid (C18:2) and the saturated stearic acid (C18:0), respectively, were responsive only to single drought in both genotypes, showing consistent declines (that were maintained at 37°C/28°C and 42°C/30°C for C18:2). By Rec14, the values from SWD plants approached those of the initial control conditions.

Finally, the moderately unsaturated *cis*+*trans* palmitoleic (C16:1*c+t*) and oleic (C18:1) acids differed from the rest of the FAs, with a negligible response to single SWD in both genotypes. With single heat, C16:1*c+t* declined in CL153 and C18:1 rose in both genotypes, but both FAs increased under stress combination (SWD, 42°C/30°C) when compared with their respective WW plants at 42°C/30°C, especially in Icatu. By Rec14, the values of C16:1*c+t* (Icatu) and C18:1 (both genotypes) were higher than under initial control conditions.

## 4 Discussion

### 4.1 Dehydration under harsh drought and/or heat conditions

Values of *Ψ*_pd_ ≤ −3.5 MPa reflect extreme water deficits (Pinheiro et al., 2004; Semedo et al., 2021), clearly below −2.15 MPa, causing leaf wilting in coffee plants (Santos and Mazzafera, 2012). Also, temperatures above 37°C (or even 39°C) exceeded thermal tolerance in *Coffea* spp (Rodrigues et al., 2016), impairing physiological, biochemical, and molecular functions (Rodrigues et al., 2016; Dubberstein et al., 2020; Marques et al., 2021). Therefore, a severe drought degree was imposed in SWD plants at 25°C/20°C, with *Ψ*_pd_ values of ca. −3.7 to −3.8 MPa, further confirmed by the strong declines of RWC_pd_ to values close to 58% in CL153 and even lower (47%) in Icatu (**Table 1**). With stress co-occurrence, the *Ψ*_pd_ values of SWD plants were further reduced to ca. −4.5 MPa at 42°C/30°C, regardless of genotype, with RWC decreasing below 50% in Icatu, thus allowing to pinpoint genotype- and stress-dependent responses of selected genes, proteins, and chloroplast membrane FAs.

Notably, by Rec14 it was observed an almost full recovery of *g*_s_ (Dubberstein et al., 2020), in agreement with the resumption of RWC_pd_ and *Ψ*_pd_ (**Table 1**), although with a tendency to lower values of these parameters in the SWD plants of both genotypes by Rec14. This suggests a considerable tolerance of hydraulic traits under harsh conditions of combined water deficit and heat and is in line with the full recovery of the same parameters after 1-week recovery to drought and cold stress in coffee genotypes, including Icatu (Ramalho et al., 2018). In fact, dehydration sensitivity has been ascribed to a lack of full recovery in water potential and stomatal conductance, accompanied by intense leaf shedding (Martins et al., 2019), none of which occurred in our study, since leaf senescence was negligible (if any) and none of the plants died during the stress period or in the subsequent recovery period. Indeed, under harsh drought under field conditions, coffee plants can tolerate extreme drought (*Ψ*_pd_ values as low as −4.4 MPa) with no plant mortality (Martins et al., 2019). This high tolerance to dehydration was associated with irrelevant (or absent) hydraulic conductivity failure and xylem vulnerability to embolism (Martins et al., 2019) and in line with the findings of Pereira et al. (2016), who predicted that coffee plant death will occur at *Ψ*_w_ as low as −7 to −8 MPa.

### 4.2 Gene transcripts and protein abundances supporting stress resilience

Coffee plants can activate a range of defense mechanisms in a species-/genotype-dependent manner (Ramalho et al., 2018; Dubberstein et al., 2020; Semedo et al., 2021). These responses support plant resilience to drought and heat and involve the synthesis and regulation of proteins that protect cellular structures, stabilize membranes, scavenge ROS (e.g., HSPs, DHNs, antioxidant enzymes), regulate water transport across cell membranes, and help maintain cellular water balance (AQPs) (Araújo et al., 2019).

#### 4.2.1 Aquaporins

AQPs are transmembrane channel proteins that increase the permeability and facilitate trafficking across biological membranes (Groszmann et al., 2016; Ahmed et al., 2021). Plasma membranes (PIPs) and tonoplasts (TIPs) are important AQP subfamilies that collectively have been reported as highly responsive to drought (Patel and Mishra, 2021), including in *Coffea* spp (Miniussi et al., 2015; Avila et al., 2020; Yaguinuma et al., 2021). PIPs have the potential to improve plant water relations and photosynthesis (Groszmann et al., 2016), whereas the presence of PIP1, PIP1,1, PIP2,1, PIP2,5, PIP2,6, PIP2,7, TIP1,2, and TIP4,1-1, was related to enhanced antioxidant defense system, reduced ROS accumulation, and decreased lipid peroxidation (Patel and Mishra, 2021) in several plant species.

Here, the abundance of gene transcripts (**Figure 1**; **Supplementary Table S2**) and proteins (**Figure 2**; **Supplementary Table S3**) of several AQPs exhibited different patterns of response to single SWD exposure, varying also between coffee genotypes in terms of protein presence, with some exceptions. In both genotypes, *CaPIP2* and *CaTIP2* were strongly downregulated under SWD (irrespective of temperature) and were largely insensitive to heat stress (both at 37°C/28°C and 42°C/30°C). This expression pattern of leaf AQPs contrasted with the abovementioned reports in other species but was in line with large transcriptional declines of *PiP1.3*, *PIP2.1*, *PIP2.4*, *PIP2.8*, and *PIP2.9* in tomato leaves and of most of the studied 35 AQP genes (PIPs and TIPs) in *Arabidopsis* (Yaguinuma et al., 2021) under drought. In addition, *PIP* and *TIP* isoforms had lower expression levels in a drought-tolerant genotype than in a sensitive one in common bean (Zupin et al., 2017).

In *C. canephora*, *CcPIP1.2* was mostly unresponsive, but CcPIP (*CcPIP2,3*, *CcPIP2,4*) and CcTIP (*CcTIP1.2*, *CcTIP2,1*) genes were downregulated in the drought-tolerant genotype (CL14) and upregulated in the drought-sensitive CL109A, indicating a genotype-dependent AQP response under severe drought (Yaguinuma et al., 2021). This aligned with the reduced expression of *CcPIP2* and *CcTIP2*, considering the high physiological resilience of Icatu and CL153 to mild and severe drought (Dubberstein et al., 2020; Semedo et al., 2021; Rodrigues et al., 2024). Accordingly, in *C. arabica* cv. Pacamara under mild drought (*Ψ*_midday_ ca. −1.3 MPa), TIP genes (*CaTIP1.1*, *CaTIP1.2*, *CaTIP4.1*) were mostly unresponsive. Yet, PIP genes *(CaPIP1.2*, *CaPIP2.1*, *CaPIP2.2*) showed expression declines that correlated with reduced leaf hydraulic conductance (*K*_Leaf_) and *Ψ*, showing that these AQPs play a role in hydraulic conductance (Miniussi et al., 2015). In fact, AQPs are constitutively expressed to maintain water homeostasis under changing water availability (Patel and Mishra, 2021). They notably affect root water transport properties (e.g., regulation of hydraulic conductance, root architecture, phloem loading, xylem water exit, nutrient acquisition), stomatal aperture, modulation of abiotic stress-related genes, and post-drought recovery (Avila et al., 2020; Li et al., 2021; Patel and Mishra, 2021). Actually, the downregulation of specific *AQP*s (Zupin et al., 2017) favors a reduction of water loss and supports leaf turgor during drought (Alexandersson et al., 2010), despite the strong dependence of transcriptional profiles of *AQP*s on the isoform, plant tissue, and stress level (Yaguinuma et al., 2021). Also, distinct regulation of different *AQP*s allows plants to switch from an anisohydric strategy (maximizing net C-assimilation and growth) under mild drought to a marked isohydric (strategy favoring water saving at the expense of productivity) under severe drought (Miniussi et al., 2015), thus contributing to acclimation and growth of crop plants during drought (Ahmed et al., 2021).

Interestingly, in our study, the mRNA abundance of *AQP*s did not consistently match with the abundance of their corresponding proteins and varied among subfamily members, stress conditions, and genotypes, demonstrating the need to accurately unveil the role of these proteins in stress response. Some AQP isoforms have their transcription level and protein abundance increased to facilitate water transport, whereas others are downregulated, reducing membrane water permeability and, thus, avoiding excessive water loss during drought exposure (Zargar et al., 2017; Yaguinuma et al., 2021). Notably, PIP proteins (PIP1.2, PIP2, PIP2.1, PIP2.7) showed an overall increase under single stresses in both genotypes, with PIP1.2 and PIP2 particularly abundant in Icatu in all stress conditions and during recovery. This would enhance stress tolerance, mainly in Icatu, as *PdPIP1.2* was associated with increased biomass, water content, and ion homeostasis (Patankar et al., 2019) and *TsPdPIP1.2* was associated with improved survival, relative water content, and lower lipoperoxidation (Wang et al., 2014) under drought. Also, *PIP2* overexpression enhanced the antioxidant defense, reduced ROS prevalence, and was linked to greater PSII maximal photochemical efficiency (*F*_v_/*F*_m_), chlorophyll content, photosynthetic rate, and water uptake (Patel and Mishra, 2021). Then, a greater abundance of these AQPs in Icatu aligned with its better PSII functioning and low chronic photoinhibition under SWD than in CL153 and similar performance up to 39°C (Dubberstein et al., 2020; Semedo et al., 2021; Rodrigues et al., 2024).

Among TIPs, TIP1.1 protein abundance increased under single drought in CL153 plants, but Icatu plants consistently tended to have higher values in all stress conditions (almost tripled in SWD-42°C/30°C) and recovery. This mirrors the findings associating *TIP1* overexpression with greater stomatal movement and leaf gas exchanges and upregulation of ROS scavenging enzymes (Patel and Mishra, 2021). Also, *PeTIP4.1–1* overexpression was associated with decreased lipoperoxidation and higher *F*_v_/*F*_m_, non-photochemical quenching (NPQ), photosynthetic functioning, and gene expression of antioxidant enzymes, namely, SOD and CAT (Sun et al., 2017; Patel and Mishra, 2021). Still, TIP4.1 was largely undetected in our plants, supporting observations of highly variable and opposite *TIP* isoform regulation depending on genotype and stress intensity (see Patel and Mishra, 2021).

In short, the protein abundance of most studied AQPs tended to increase in response to drought (e.g., PIP2 and PIP1.2 in Icatu; TIP1.1 in both genotypes), likely preserving hydraulic traits in these genotypes (as mentioned in 4.1). Icatu was more responsive, but CL153 seemed to present a higher constitutive level of these AQPs. In addition, PIP2, PIP1.2, and TIP1.1 showed increased protein abundance under heat only in Icatu, which is globally in accordance with these genotypes’ physiological resilience to water and/or heat stresses (Dubberstein et al., 2020; Semedo et al., 2021; Rodrigues et al., 2024). In addition, although AQPs are usually associated with dehydration, the observed heat response in Icatu-WW plants was unrelated to a possible leaf dehydration given that their water status was little, if at all, altered along the temperature rise [as assessed by the almost invariant RWC_pd_ and *Ψ*_pd_ values (**Table 1**)], despite the large increase in the transpiration flow (Dubberstein et al., 2020).

#### 4.2.2 Dehydration-Responsive Element Binding transcription factor

Under adverse conditions, signaling (TF) functions are crucial to maintain cell homeostasis (Balfagón et al., 2020). *DREB1D-F1*, a drought-responsive transcription factor, was markedly upregulated in both genotypes under drought and was further enhanced under stress superimposition (at 37°C/28°C and 42°C/30°C) in CL153 (**Figure 1**; **Supplementary Table S2**), likely associated with the intrinsic physiological stress resilience of these genotypes (Dubberstein et al., 2020; Rodrigues et al., 2024). In fact, the upregulation of DREB-like genes (e.g., *CcDREB1D*, *CcDREB1B*, *CcRAP2.4*, *CcERF027*) was associated with drought tolerance in *C. canephora* clones (Marraccini et al., 2012; Torres et al., 2019; Thioune et al., 2020) through an ABA-dependent pathway (Torres et al., 2019), being responsive also to drought and high and low temperatures in the leaves of *C. arabica* (Alves et al., 2018; Torres et al., 2019).

#### 4.2.3 Molecular chaperones—DHNs, HSP70, ELIP, Chape 20, and Chape 60

Plant chaperones play key roles in protein protection in both optimal and adverse conditions (Wang et al., 2004), being closely modulated by stress stimuli, namely, temperature and drought. Among them, DHNs are a multifunctional and diverse class of proteins that are involved in biomolecule and membrane stabilization and protection against lipoperoxidation, due to their antioxidative activity as a free radical scavenger (Theocharis et al., 2012; Gupta et al., 2019; Tiwari and Chakrabarty, 2021; Szlachtowska and Rurek, 2023). In addition to their chaperone role, DHNs participate in the cell transcription regulatory machinery, regulating stress-responsive genes and epigenetic processes (Tiwari and Chakrabarty, 2021). The increase of DHN abundance has been associated with improved heat tolerance through the maintenance of membrane integrity in sugarcane (*Saccharum officinarum*) seedlings (Szlachtowska and Rurek, 2023), whereas the accumulation of *DHN1* transcripts in olive tree (*Olea europaea*) increased after heat and, especially, during drought exposure (Araújo et al., 2019). In our coffee plants, *DH1a* transcription was mostly unresponsive in CL153 but was strongly induced in all conditions in Icatu (including Rec14), likely promoting membrane stabilization and ROS scavenging (**Figure 1**; **Supplementary Table S2**). This partly agrees with transcript accumulation of several *DHNs* in the roots and leaves of *C. arabica* cvs. Catuaí and Mundo Novo and *C. canephora* cv. Apoatã under mild drought. *CaDHN1* and *CaDHN3* were constitutively expressed, whereas *CaDHN2* was exclusively expressed in stressed plants, showing that *DHNs* are involved in *Coffea* spp. response to drought, with different temporal and extent expression levels among genes (Santos and Mazzafera, 2012). Despite the distinct transcription pattern of *DH1a* in our genotypes, the DH1a protein greatly responded to drought at all temperatures, but not to single heat (thus, suggesting a specific response to drought, although with a positive interaction in CL153 at 37°C/28°C), and returned to control values by Rec14 (**Figure 2**; **Supplementary Table S3**). This rise of DH1a abundance occurred with the unaltered lipoperoxidation of SWD plants from 25°C/20°C (single drought) up to 37°C/28°C (Rodrigues et al., 2024), likely with the DHNs’ protective contribution associated with the electrostatical “cross-linking” of membrane lipids (Gupta et al., 2019).

HSPs constitute another chaperone family associated with stress response. They have multiple functions, assisting protein folding and preventing irreversible protein aggregation (Wang et al., 2004; Park and Seo, 2015; Cheng et al., 2015; Wang et al., 2018), facilitating the translocation and degradation of unstable proteins (Wang et al., 2004; Fragkostefanakis et al., 2015), and acting as ROS sensors (e.g., H_2_O_2_), in addition to controlling the expression of oxidative stress response genes during oxidative stress (Miller and Mittler, 2006; Volkov et al., 2006). HSP70 has been associated with the reestablishment of cellular homeostasis and crop protection and resilience against environmental constraints (Wang et al., 2004) such as heat (Lamaoui et al., 2018) and drought (Park and Seo, 2015; Wang et al., 2018). Consistent with its role in protein homeostasis, thermotolerance, and PSII protection, single drought tripled the abundance of the HSP70 protein, in line with the high expression of several HSP genes in *C. canephora* in response to drought (Thioune et al., 2020) and with *HSP70* expression in *C. canephora* and *C. arabica* under mild (Fernandes et al., 2021; Marques et al., 2022b) and severe water deficit (Marques et al., 2022b; Rodrigues et al., 2024). However, heat was the major driver of *HSP70* transcription and protein abundance, with protein increasing under single 42°C/30°C (up to 4.5-fold) or combined with SWD, especially in Icatu (with maximal values up to 7-fold higher), keeping considerable values by Rec14. These findings agree with the strong upregulation of HSP70 genes in response to temperatures from 31°C (Martins et al., 2016) up to 42°C (Vinci et al., 2022) in *C. arabica*, likely contributing to leaf resilience and recovery (Rodrigues et al., 2024).

ELIPs are low molecular mass stress proteins, belonging to the multigenic family of light-harvesting complexes (LHC) from thylakoid membranes (Adamska, 2001; Hutin et al., 2003). They accumulate transiently upon high irradiance, binding to free chlorophyll molecules, preventing the formation of free radicals, and promoting the stabilization of chlorophyll, LHC, and photosystems at the thylakoid level and/or by acting as sinks for excitation energy, thus protecting chloroplasts from photooxidative stress (Adamska, 2001; Hutin et al., 2003), promoted by drought (Kwon et al., 2021) or cold (Montané et al., 1997). Here, drought prompted a large accumulation of *ELIP* transcripts and protein in Icatu, likely assisting the reported resilience of PSII electron transport activity (Semedo et al., 2021), photochemical efficiency (*F*_v_/*F*_m_) (Dubberstein et al., 2020), and lower chronic photoinhibition (Rodrigues et al., 2024). Also, *ELIP* responsiveness (but not protein abundance) was even stronger under single heat conditions in both genotypes, but only Icatu showed a positive stress interaction, with maximal transcripts and protein abundance in SWD plants at 42°C/30°C. Although this was scarce to ensure full PSII protection at the harshest conditions (Dubberstein et al., 2020; Rodrigues et al., 2024), it is worth mentioning that Icatu-SWD plants showed lower PSII impact than WW counterparts at 42°C/30°C. Overall, ELIPs likely contributed to preserve PSII function in *Coffea* spp. under drought and especially heat and stress interaction (42°C/30°C), with a response to heat and stress interaction that, to the best of our knowledge, has never been described.

Chaperonins belong to the “foldases group” of proteins (Askari-Khorasgani and Pessarakli, 2019), with 20 chaperonins from chloroplasts and 60 from the mitochondria. Chaperonins are protective molecules associated with stress tolerance (e.g., against heat), by assisting a wide range of newly synthesized and newly translocated proteins to achieve their assembly and native forms, namely, RuBisCO (see Wang et al., 2004) and chloroplast ATP synthase coupled to thylakoid electron transport (Iba, 2002; Ahn and Zimmerman, 2006; Mao et al., 2015). The upregulation of *Chape 20* and *Chape 60* under drought was reported in *Coffea* spp (Guedes et al., 2018; Fernandes et al., 2021), in line with moderate upregulation of *Chape 20* and *Chape 60* in both genotypes in response to SWD (significant only for *Chape 20* in CL153), together with a tendency to higher abundance of both proteins in both genotypes (significant for Chape 60 in Icatu). However, maximal protein abundance levels were found at 37°C/28°C (Chape 20) and at 42°C/30°C (Chape 60), with a stronger increase found for Chape 60 in Icatu. Thus, our findings highlighted heat as a greater response driver than drought of these gene transcripts and protein abundance levels, confirming the reports of *Chape 20* and *Chape 60* being responsiveness to heat in *Coffea* spp (Martins et al., 2016; Vinci et al., 2022). Also, as chloroplastic chaperonin cofactor (CPN20) can mediate FeSOD activity (Kuo et al., 2013), the increase of Chape 20 might have contributed to a lesser inactivation status of PSII and the preservation of thylakoid electron transport under drought (Semedo et al., 2021) and heat (Martins et al., 2016) in CL153 and Icatu.

#### 4.2.4 Antioxidative and photoprotection-related enzymes

Photoinhibition of photosynthesis often occurs when absorbed light energy by LHCII pigments exceeds the capability for its photochemical use (Nishiyama and Murata, 2014; Tikkanen and Aro, 2014), promoting oxidative stress as a secondary stress. To prevent ROS formation, plants employ mechanisms of energy dissipation (e.g., through pigments such as zeaxanthin and lutein) and thermal dissipation (e.g., via non-photochemical quenching). However, upon ROS overproduction, efficient scavenging becomes essential to prevent oxidative damage, maintaining cellular homeostasis, which is achieved by enzyme and non-enzyme antioxidative components acting complementarily. In short, superoxide radicals (O_2_^○−^), produced when O_2_ is the electron acceptor from thylakoid electron transport (particularly at the PSI level), are converted by Cu/Zn-SOD into hydrogen peroxide (H_2_O_2_). This toxic molecule must then be neutralized prior to its conversion into a highly reactive hydroxyl radical (OH^○^) by APX (with ASC) and catalase (Logan, 2005; Smirnoff, 2005; Wang et al., 2018).

In *Coffea* spp., the protective mechanisms mentioned above are triggered in response to single environmental constraints, such as high irradiance (Ramalho et al., 1998), drought (Lima et al., 2002; Ramalho et al., 2018; Semedo et al., 2021), cold (Ramalho et al., 2003, 2014; Batista-Santos et al., 2011), and heat (Martins et al., 2016; Rodrigues et al., 2016; Vinci et al., 2022). In addition, genes associated with tolerance to drought in *C. canephora* (Vieira et al., 2013; Guedes et al., 2018) and *C. arabica* (Mofatto et al., 2016; Fernandes et al., 2021) and, to a lower extent, to heat (Martins et al., 2016; Marques et al., 2021) include those related to ROS control (e.g., *CuSOD* and *APX*). This agrees with the great abundance responsiveness of all the studied enzymes (CAT, APX Cyt, APX Chl, APXt+s, and VDE) to SWD (except Cu/ZnSOD_2_) and to heat (except Cu/ZnSOD_2_ and VDE) in both genotypes, with Icatu showing a further increase of APX proteins under stress superimposition (**Figure 2**; **Supplementary Table S3**). *CuSOD_2_* transcription slightly responded to drought or heat, with maximal values under their combination in both genotypes (**Figure 1**; **Supplementary Table S2**), in line with the moderate changes in protein abundance. However, Cu/ZnSOD activity greatly increased under SWD in CL153 and Icatu (Rodrigues et al., 2024), evincing the need to neutralize the produced H_2_O_2_ (e.g., by Cu/ZnSOD and photorespiration) through APX and CAT action. In fact, CAT abundance largely increased due to drought or heat (also in Rec14), particularly in Icatu. This finding aligns with CAT responsiveness to cold (Fortunato et al., 2010), drought (Ramalho et al., 2018), and heat (Vinci et al., 2022) in *Coffea* spp., typically showing a higher activity in *C. arabica* than in *C. canephora* genotypes (Rodrigues et al., 2024). Yet, the higher CAT abundance in Icatu at 42°C/30°C paralleled a significant activity decline (Rodrigues et al., 2024).

Still regarding H_2_O_2_ removal, some APX isoforms were among the most stress-responsive components to both SWD and heat, demonstrating their key role in the antioxidative system of *Coffea* spp. under drought and/or heat. Strong gene upregulation was observed in *APXCyt* in CL153 and in *APXChl* in Icatu in response to drought, in all APX genes in response to heat (in both genotypes, but especially *APXCyt* in CL153), and in all APX genes in Icatu under stress superimposition. Furthermore, APX abundance was among the most responsive to drought and heat in both genotypes and under stress superimposition in Icatu. Peroxisomal APX isoform was less responsive in CL153 than in Icatu in all stress conditions, both in transcripts and protein abundance, thus reinforcing the importance of isoform–genotype responses in stress acclimation. Also, APXChl abundance was highly responsive to all stress conditions in Icatu, especially under stress superimposition (and partly maintained by Rec14) that would have granted the plants a strong potential for H_2_O_2_ removal in the chloroplast. This global APX responsiveness (regarding both transcript and protein abundance) agrees with APX activity under drought (Ramalho et al., 2018) and heat (up to 37°C) (Martins et al., 2016; Vinci et al., 2022; Rodrigues et al., 2024), usually with a lower response in *C. canephora* (Marraccini et al., 2012). However, as for CAT, a mismatch between APXChl protein abundance and enzymatic activity (Rodrigues et al., 2024) occurred in Icatu SWD plants at 42°C/30°C. For both CAT and APXChl, the decline of activity paralleled their greater protein abundance at 42°C/30°C. This suggests a degree of thermal sensitivity of these enzymes at 42°C/30°C that could compromise H_2_O_2_ removal, thus aligning with the increase in lipoperoxidation and loss of membrane selectivity and photosynthetic performance in both genotypes (Rodrigues et al., 2024). Also, it highlighted the risk of relying solely on transcripts and protein abundance data when evaluating protective capacity.

VDE controls zeaxanthin (Zea) synthesis, a photoprotective liposoluble pigment from the light-harvesting complexes (*LHCs*). Zea scavenges ^1^O_2_; thermally dissipates the excess of light energy, thus reducing the formation of highly reactive molecules of Chl (^3^Chl* and ^1^Chl*); and acts against the photooxidation of membrane lipids by removing epoxy groups from the oxidized double bonds of FAs of chloroplast membranes (Havaux and Niyogi, 1999; Adams et al., 2002; Dall’Osto et al., 2012). Zea photoprotects the photosynthetic machinery of *Coffea* spp. against drought (Ramalho et al., 2018; Rodrigues et al., 2024) but is mostly unresponsive to heat (Martins et al., 2016). This agrees with the large increase in VDE abundance (but not gene expression) upon single SWD, especially in Icatu, supporting the higher Zea content and de-epoxidation state (Rodrigues et al., 2024). Also, only Icatu showed a global increase of the xanthophyll cycle pool components and a larger lutein value, altogether contributing to control lipoperoxidation under SWD (Rodrigues et al., 2024). In contrast, the VDE transcripts and protein abundance barely responded to heat, which aligned with the absence of significant Zea rise at any supra-optimal temperature (Rodrigues et al., 2024), suggesting a thermal lability of VDE that would limit Zea synthesis. This would additionally contribute to a strong ASC decline (Martins et al., 2016), as Zea is involved in ASC regeneration (Logan, 2005; Smirnoff, 2005). This would limit APX function and negatively impacted photosynthetic functioning and stress tolerance (Rodrigues et al., 2024), both of which affect yield. Interestingly, VDE protein increased in Rec14 in Icatu (without *VDE* upregulation), with greater Zea and ASC contents in Icatu-SWD plants, pointing to the need for photoprotective reactivation 2 weeks after the end of stress exposure.

Altogether, the above findings clearly highlighted the upmost importance of a coordinated action of antioxidative components under drought and/or heat to effectively safeguard the photosynthetic machinery. Despite the reinforcement of several protective molecules (e.g., PIPIs, DH1a, HSP70, ELIP), the ability to acclimate at the highest temperature (but not of SWD) was compromised by thermal sensitivity (42°C/30°C) of some oxidative stress control components, namely, of CAT, APXs, and VDE (and Zea), despite their greater protein abundance. Also, the modest correlations between transcriptomic, proteomic, and physiological data noted in *Coffea* spp. here and elsewhere (Fernandes et al., 2021; Marques et al., 2023) point to the presence of regulatory mechanisms other than transcriptional and the need to integrate several levels of complementary analysis to obtain an accurate perspective of plant performance and yield stability in *Coffea* spp. facing climate-related stressors. Genotypic differences in these responses (particularly Icatu resilience) offer valuable traits aiming at breeding climate-resilient coffee cultivars.

### 4.3 Chloroplast membrane lipid dynamics under stress

Unlike CL153, which showed minimal quantitative changes in TFAs in response to drought and/or heat, Icatu exhibited marked lipid *de-novo* synthesis under drought, moderate heat (37°C/28°C) and, especially, under stress combination (SWD-37°C/28°C) (**Table 2**). This greater responsiveness of the *C. arabica* genotype reflects greater lipid metabolic plasticity, a key to stress acclimation to cold (Partelli et al., 2011; Scotti-Campos et al., 2014) and heat (Scotti-Campos et al., 2019). Such lipid metabolism flexibility was further reflected in important qualitative changes. The reprogramming of the FA profile occurred under drought (both genotypes) or heat (only in Icatu, up to 37°C/28°C), through an increase in the unsaturation level (reflected in DBI rise). This shifted the balance between the two most represented FAs, C16:0 and C18:3, which were reduced and increased, respectively. Additionally, single heat (42°C/30°C) increased C16:0 in CL153. Such drought- and/or heat-prompted (37°C/28°C) unsaturation (greater in Icatu) would support membrane fluidity and functionality (Gombos and Murata, 1998; Routaboul et al., 2000). This is highly relevant for maintaining C-assimilation, since photosynthetic performance closely depends on higher FA unsaturation to preserve lipid acyl motion in thylakoid membranes (Harwood, 1998; Siegenthaler and Tremolieres, 1998), concerning PSI and PSII thylakoid electron transport rates, which are membrane-based events. These traits are also crucial to integrate newly synthesized D1 (Kern and Zouni, 2009) related to the repair processes needed to sustain PSII function under stress. This agrees with the preservation of the photosynthetic machinery potential in *Coffea* spp. under SWD (Dubberstein et al., 2020; Semedo et al., 2021) and up to 37°C/28°C, but with impacts at 42°C/30°C (Dubberstein et al., 2020; Rodrigues et al., 2024), when DBI falls as compared with 37°C/28°C. Still, a higher abundance of polyunsaturated FAs also increases lipoperoxidation risk, as double bonds are preferential targets of hydrolytic enzymes, peroxidases, ROS, and free radicals (Girotti, 1990; Öquist, 1982), thus requiring a complementary strengthening of antioxidative defenses, as reported under cold (Fortunato et al., 2010) and heat (Martins et al., 2016). That was the case here under SWD and heat (37°C/28°C), since although with different extent, both genotypes, particularly Icatu, showed an enhanced coordination of lipid matrix remodeling together with greater abundance of antioxidant enzymes (APXs, CAT) and their activity (Rodrigues et al., 2024), stress proteins (HSP70, Chaperonins), and membrane protectors, such as AQPs (PIP1.2, PIP2.1, PIP2.7, TIP1.1) and DH1a, all contributing to membrane protection. For instance, the presence of several PIPs (PIP1, PIP1,1, PIP2,1, PIP2,5, PIP2,6, PIP2,7) and TIPs (TIP1,2, TIP4,1-1) was associated with enhanced antioxidant capability and lowered ROS presence and lipid peroxidation level (Patel and Mishra, 2021), whereas DHNs, which bind to membrane lipids, have antioxidative activity, and their accumulation protects membrane integrity, acting against lipoperoxidation (Theocharis et al., 2012; Tiwari and Chakrabarty, 2021; Szlachtowska and Rurek, 2023).

Notably, only Icatu increased C16:1*c*+*t* values (due to greater TFA abundance) under single drought, stress interaction (SWD, 42°C/30°C), and Rec14 (WW plants), likely contributing to chloroplast membrane stability (Scotti-Campos et al., 2014). C16:1*t* is an exclusive component of thylakoid phosphatidylglycerol (PG) (Öquist, 1982; Siegenthaler, 1998), and both PG and C16:1*t* are involved in the supramolecular thylakoid membrane organization of LCHII proteins and pigments, stabilizing the PS complexes and allowing an efficient non-cyclic electron flow (Siegenthaler and Tremolieres, 1998; Vijayan et al., 1998; Yang et al., 2005). This FA is also involved in the process of replacement of damaged D1 protein (Siegenthaler and Tremolieres, 1998; Harwood, 1998; Gray et al., 2005), reducing PSII photoinhibition and promoting a faster recovery from the photoinhibited state (Gombos and Murata, 1998; Siegenthaler and Tremolieres, 1998), in accordance with Icatu resilience and recovery under drought (Semedo et al., 2021) and heat (Dubberstein et al., 2020).

Overall, the ability to remodel the lipid matrix of chloroplast membranes is a crucial feature to drought/heat resilience in *Coffea* spp., working in tandem with complementary protection mechanisms (e.g., antioxidative), ultimately sustaining productivity under adverse conditions.

### 4.4 Early-stage biomarkers for stress resilience screening in *Coffea*

Accelerating the identification of drought- and heat-resilient genotypes is a key objective in breeding programs, especially under the pressing challenges of climate change. Traditional phenotyping approaches rely on the evaluation of adult plants in field conditions, which are time-consuming, highly variable, and resource-intensive. Our findings suggested that several stress-responsive traits (at the molecular, biochemical, and physiological levels) are detectable in the leaves, providing a potential shortcut for selecting resilient genotypes. Also, they reinforced the possibility of using environmental controlled stress experiments to assess genotypic plasticity during the vegetative phenophase. Integrating gene expression (e.g., DREB1D-F1, APXChl), enzyme protein abundance and activity (e.g., CAT, APX), and lipidomic profiling and unsaturation of FAs (e.g., DBI, C16:0, C18:3, C16:1*t*) could form the basis of high-throughput screening tools for the pre-field selection of elite genotypes.

Future studies should focus on validating the here identified biomarkers across developmental stages and diverse genetic backgrounds, defining threshold expression or activity levels that reliably predict long-term stress performance, developing cost-effective protocols (e.g., qPCR panels, ELISA kits, targeted omics) applicable in breeding nurseries and controlled environments. Still, it is noteworthy that gene transcripts and corresponding protein abundance and activity often showed a distinct pattern of response, pointing to the need for an integrated proteomic, transcriptomic, and ecophysiological analysis to get a truly accurate perspective. Overall, the identification of these reliable stress-responsive traits opens new avenues for accelerating the selection of drought- and heat-resilient *Coffea* spp. cultivars, reducing breeding cycles and enhancing genetic gain under climate stress scenarios.

## 5 Conclusions

Overall, severe drought is a greater response driver of most defense mechanisms than heat (37°C/28°C and/or 42°C/30°C). Drought might even act as a priming factor to heat, with the drought responses being maintained or even incremented (interaction) under simultaneous exposure to both stresses (**Supplementary Table S4**).

Specific responses associated with severe dehydration included a strong upregulation of *DREB1D-F1* (both genotypes) and *APXChl* (Icatu), along with increased abundance of some proteins, such as DH1a, ELIP, CuSOD2, and VDE (both genotypes) and TIP1.1 and APXt proteins (CL153). Lipid remodeling (decline of C16:0 and increase of C18:3 and DBI) was also found in CL153 just for SWD. In contrast, heat triggered few specific responses, with the upregulation of *Chape 20* (Icatu) and *VDE* (CL153) and increased abundance of PIP2.1 (both genotypes) and PIP2.7 (CL153). Increases of C16:0 (CL153) and C18:1 (both genotypes) reflected limited membrane adjustments as compared to drought.

A broad and robust response was commonly triggered to both single stresses, mainly in Icatu. This included greater numbers of upregulated genes and stress-responsive proteins, such as *DHA1* transcripts, and PIP2, PIP1.2, and TIP1.1 protein abundance increased only in Icatu (despite the downregulation of *caPIP2*), together with *ELIP* (although protein abundance increased only under drought), *HSP70*, *Chape 20*, and *Chape 60* (greatly with heat) in both genotypes. Particularly, a strong upregulation of *APXCyt* (CL153) and *APXChl* (both genotypes) and increases in protein abundance also for CAT, APXCyt, APXChl, and APXt+s were found (always greater or only in Icatu), altogether underscoring the importance of integrated ROS control and membrane protection in stress acclimation. Increases in TFA, 18:3, and DBI, in parallel with C16:0 decline (by 37°C/28°C), were observed only in Icatu, supporting membrane flexibility and photosynthetic function.

Drought and heat stress crosstalk was evident in a few cases, especially in Icatu, which showed additive or synergistic responses, with the upregulation of several genes (*HSP70*, *ELIP*, *Chape 60*, and all APXs—*APXCyt*, *APXChl*, *APXt+s*), as well as increased protein abundances (PIP2, TIP1.1, ELIP, and APXChl) and TFA content (at 37°C/28°C). In contrast, only *Chape 20* and *DH1a* denoted specific CL153 stress interaction. In addition, both genotypes displayed *DREB1D-F1* and *CAT* upregulation, together with further increases in DH1a protein and C16:1*c+t* and C18:1 (especially Icatu), as compared with the WW plants under 42°C/30°C, denoting common lipid response dynamics. Notably, the response of AQPs and ELIP to heat (thus unrelated to dehydration) and/or stress interaction has never been described before at the chloroplast membrane level.

Relevant recovery capacity was found in both genotypes after stress relief (Rec14), with the sustained expression of key defense genes, such as *DREB1D-F1*, *APXCyt*, *Chape 20* (all greater in CL153), *ELIP*, *DH1a HSP70*, and *Chape 60*, and of protein abundance, such as Chape 20 (greater in CL153), APXCyt and CAT (both greater in Icatu), Chape 60, and APXChl. Icatu specifically maintained an upregulation of *APXt+s*, as well as increased abundance of proteins (PIP2, PIP1.2, TIP1.1, APXt+s) and TFA, suggesting superior post-stress adjustment that could improve plant resilience to subsequent stress events.

In summary, a core set of complementary protective mechanisms was associated with drought and/or thermal tolerance. Drought is usually a greater driver of plant responses than heat, but a number of responses are commonly triggered under both stresses and by their interaction. Changes include remodeling in the lipid chloroplast membrane matrix, integrated with the strengthening of protective and oxidative stress control mechanisms, namely, via AQPs, DHN1, HSP70, chaperonins, VDE, and antioxidative enzymes (CAT and especially APXs). These mechanisms are expressed either commonly or in a stress-specific or genotype-dependent manner, but a broader and more effective response (in the number of genes/molecules and response degree) is usually found in Icatu, including after stress relief, in line with its resilience under drought and heat reported previously at the physiological level. These constitute key insights to ensure this crop’s future sustainability, used to accurately select and breed resilient genotypes better suited to climate changes ahead that will surely include a higher frequency of combined drought and heat events.

## Data Availability

The mass spectrometry proteomics data have been deposited to the ProteomeXchange Consortium via the PRIDE partner repository with the dataset identifier PXD019474 and DOI: 10.6019/PXD019474 for *C. arabica* proteome, and the dataset identifier PXD019541 and DOI: 10.6019/PXD019541 for *C. canephora* proteome.
