# Supplementary material for: Stress resilience in Coffea arabica and Coffea canephora under harsh drought and/or heat conditions: selected genes, proteins, and lipid integrated responses
Source: Front Plant Sci. 2025 Jul 24;16:1623156. doi: 10.3389/fpls.2025.1623156 (PMC12328417; doi:10.3389/fpls.2025.1623156)
Supplement: Supplementary file 1 [file Supplementaryfile1.docx]

**Supplementary Table S1**. Selected genes used for real-time qPCR studies (which are related to protective proteins), homologies, and primer sequences, and the original reference from where they were obtained.


**Supplementary Table S2**. Transcription studies of selected genes associated with stress response mechanisms in *Coffea canephora* cv. Conilon Clone 153 (CL153) and *Coffea arabica* cv. Icatu, submitted to well-watered (WW) and severe water deficit (SWD), and temperature increase from (25/20 ºC, day/night), to 42/30 ºC, followed by a 14-day recovery period after stress relief (Rec14). The RT-qPCR gene expression values represent the n-fold relative to the double control (25/20 ºC, WW) within each genotype. Original expression values for each gene resulted from the mean ± SE (n=6-9), from 3 independent biological assays. For each gene, different letters after the mean values express significant differences between temperature treatments for the same water level (A, B, C, D), or between water availability levels for each temperature treatment (a, b), always separately for each genotype.

**Supplementary Table S3**. Changes in protein abundance of selected proteins associated with stress response mechanisms, assessed through the number of MS/MS spectral counts associated with each protein in *Coffea canephora* cv. Conilon Clone 153 (CL153) and *Coffea arabica* cv. Icatu plants submitted to well-watered (WW) and severe water deficit (SWD), followed by a temperature increase from (25/20 °C, day/night), to 42/30 °C, and a 14-day recovery period after stress relief (Rec14).For each protein, different letters after the mean values ± SE (n=3) express significant differences between temperature treatments for the same water level (A, B, C, D), or between water availability levels for each temperature treatment (a, b), always separately for each genotype.


**Supplementary Table S4**. Summary of the gene up/down-regulation, protein abundance and lipid components associated with defence mechanisms triggered in *Coffea canephora* cv. Conilon Clone 153 (CL153) and *Coffea arabica* cv. Icatu plants, considering specific (positive or negative) responses to 1) severe drought (SWD), or 2) heat (37/28 or 42/30 °C), 3) response interaction under the stress superimposition (SWD and heat), and 4) responses after a 14-day recovery period after stress relief (by Rec14). In the coloured cells, the “+” or "-" signal indicates the existence of positive/rise or negative/decline response, respectively, whereas the strength of response between genotypes is given by the number of “+” or "-" signals. Empty cells denoted absence of response; white with "-" signal denoted absence of results. Blue and green identify changes in CL153 and Icatu, respectively.
